# Promoting Emotional Well-Being in Older Breast Cancer Patients: Results From an eHealth Intervention

**DOI:** 10.3389/fpsyg.2018.02279

**Published:** 2018-11-27

**Authors:** Daniela Villani, Chiara Cognetta, Claudia Repetto, Silvia Serino, Davide Toniolo, Francesco Scanzi, Giuseppe Riva

**Affiliations:** ^1^Department of Psychology, Università Cattolica del Sacro Cuore, Milan, Italy; ^2^Department of Medical Oncology, G.Salvini ASST Rhodense, Milan, Italy; ^3^Applied Technology for Neuro-Psychology Lab, Istituto Auxologico Italiano, Milan, Italy; ^4^U.O. Oncologia Medica, Ospedale S. Giuseppe-Multimedica, Milan, Italy

**Keywords:** emotion regulaton, well-being, positive technology, eHealth, breast cancer

## Abstract

Breast cancer is the most common cancer in women worldwide, with increases in diagnoses at all ages. Due to several age-related factors, older breast cancer patients show particular difficulties in adjusting to breast cancer and its related treatments. One consistent indicator of vulnerability to long-term complications is emotional distress occurring within 3 months of diagnosis. Thus, it is critical to develop early interventions specifically aimed at mitigating distress and promoting emotional wellbeing in older breast cancer patients. By taking advantage of the opportunities of online interventions, the present study aimed to test the efficacy of a 2 weeks e-health stress inoculation training (SIT) intervention on emotion regulation and cancer-related well-being, compared with a control group without such intervention. Twenty-nine women with a diagnosis of breast cancer, who had received radical surgery and who were suitable candidates for adjuvant chemotherapy with anthracyclines and taxanes (mean age = 62.76; *SD* = 6.19) voluntarily took part in the current study after giving written informed consent. To test intervention efficacy, self-report questionnaires were administered to all participants at baseline, at the end of the 2 weeks intervention, and 3 months after the end of the intervention. Results showed that after 2 weeks of ehealth intervention, patients did not achieve significant change, however, they significantly reduced emotional suppression and increased cancer-related emotional well-being 3 months after the end of the intervention. Furthermore, by monitoring at a distance the emotional experience during the online intervention, we found an increase in relaxation and a reduction of anxiety. Finally, patients in the experimental group reported a good level of acceptance of the ehealth intervention. To conclude, designing and developing eHealth interventions as part of the regular care path for breast cancer patients of all ages represents both a challenge and an opportunity; in particular, online interventions can be an important step in universal psychosocial care within a tiered model of care.

## Introduction

Breast cancer is the most common cancer in women worldwide. With increases in diagnoses at all ages – even if more slowly between 50 and 80 years of age (DeSantis et al., 2011) – more women will have to deal with breast cancer and its consequences (van Ee et al., 2017b).

From a psychological point of view, the literature is not consistent about women’ experiences related to age. Although women of different ages have many experiences in common regarding breast cancer that imply a deterioration of well-being and quality of life (Montazeri, 2008; Campbell-Enns and Woodgate, 2016), older women do not have to deal with the non-normative nature of a chronic disease at a relatively young age, which typically causes disruption in multiple life roles (Dunn and Steginga, 2000; Thewes et al., 2004). They are less challenged by job demands, taking care of a young family, and fertility issues (Rana et al., 2017). Nevertheless, a variety of age-related aspects can make it more stressful for older patients to deal with the diagnosis and treatment of breast cancer (Park et al., 2011).

Several studies showed the emergence of psychosocial distress soon after the diagnosis of cancer and before the beginning of treatment (Nosarti et al., 2001; Andreu et al., 2012). Since high levels of distress during or immediately after surgery (Nosarti et al., 2001; Gallagher et al., 2002; Lam et al., 2007; Mertz et al., 2012) can lead to higher psychological vulnerability in the subsequent treatment period (Neerukonda et al., 2015), it is crucial to address this risk early on in the treatment process (Henselmans et al., 2010; Lam et al., 2012). In particular, chemotherapy treatment has been reported as distressing and traumatizing (Richer and Ezer, 2002), including for post-menopausal women (Browall et al., 2006), with hair loss, nausea and fatigue frequently ranked among the first three worst side effects (Carelle et al., 2002).

According to a recent review, emotional distress, measured within 3 months of diagnosis, is the only consistent predictor that the distress will persist long-term (Cook et al., 2018). Emotional distress is associated less with the patient’s actual clinical condition than it is to the patient’s own subjective opinion of their health and prognosis (Millar et al., 2005). The persistence of distress as an enduring problem, at least for a third of patients in treatment or long-term follow-up (Cook et al., 2018), supports the need for assessing distress and developing early interventions specifically aimed at mitigating distress and promoting individual wellbeing (Casellas-Grau et al., 2014).

In the scientific literature, attention to the effectiveness of psycho-oncological interventions for patients with breast and other cancers seems to diminish with increasing age, specifically after 60 years of age (van Ee et al., 2017a). Thus, psychological interventions designed to reduce anxiety in older women and increase their control over the treatments and treatment-related effects (Treacy and Mayer, 2000; Seçkin, 2011) and evidence-based recommendations represent a future challenge (Petrakis and Paraskakis, 2010; Biganzoli et al., 2012).

## The Opportunities of ehealth Interventions

Computer-based and web applications have demonstrated their potential for supporting psychological interventions aimed to help people cope with health-related distressing experiences. The Positive Technology approach suggests various options for using advanced technology to facilitate psychological health and wellbeing (Botella et al., 2012; Riva et al., 2012; Villani et al., 2016a). According to this approach, positive technologies can be categorized according to their effects on three features of personal experience: *hedonic*, such as technologies used to induce positive and pleasant experiences; e*udaimonic*, such as technologies used to support individuals in reaching engaging and self-actualizing experiences; and *social/interpersonal*, such as technologies used to support and improve the connectedness between individuals, groups, and organizations. Positive technology tools can be used to effectively promote patients’ clinical change; but to reach this challenge, they must adapt to the specific stage of the patients’ change process and sustain their engagement within the provided experience (Riva et al., 2016). Within the positive technology approach, eHealth interventions allow the development of sustainable and patient-centered services, alternatively focused on several experiential dimensions (i.e., cognitive, emotional or behavioral) of patient engagement related to their healthcare management (Barello et al., 2016). Over the last decade, the application of eHealth interventions in psycho-oncological care has grown (McAlpine et al., 2015), and cancer patients are becoming Internet users, independently of their age, breast cancer stage and length of time since diagnosis (Fogel et al., 2002).

The Internet has become an accessible source for individuals to research information, including for their medical issues, and patients with cancer are among the most frequent such users (Drewes et al., 2016). This reflects the development of Internet educational programs available for breast cancer patients that, at the moment, are focused mostly on increasing patients’ knowledge through information related to both the disease and the procedures (Warren et al., 2014). Clear, accurate and informative websites can be seen as a first level of supportive care, but the mere provision of educational material does not significantly improve compliance or patients’ wellbeing. Thus, it is possible to recognize eHealth interventions that are more oriented to sustaining women’s well-being (Villani et al., 2016b). In one recent study, women with cancer identified Web-based applications, email, and blogs as appropriate vehicles to meet their needs for psychological and informational support and specified their preference for topics such as ability to cope, anxiety and depressive feelings (Ringwald et al., 2017).

These interventions vary from (1) the use of personal websites aimed to enhance the emotional wellbeing of women with breast cancer through the development of narrative experiences and the expression of their feelings (Harris et al., 2015); (2) the development of online peer support interventions aimed to enhance social support, which have been appreciated by older women (Seçkin, 2011); and (3) more sophisticated training aimed at teaching self-guided coping skills principally oriented toward the management of patients’ affective state (Owen et al., 2005).

Coherently with this third approach, another opportunity is offered by adapting Meichenbaum’s stress inoculation training (SIT) (Meichenbaum, 1985) within eHealth interventions. SIT is a cognitive behavioral therapy designed to strengthen the patient’s coping strategies to deal with stress. In the breast cancer context, the aim is to prepare women for chemotherapy treatment and side effects by helping them reduce the potential negative cognitive, emotional, and behavioral reactions. The clinical rationale behind this approach is that to effectively manage stress it is crucial to “inoculate” the stressor: using a combination of graded exposure with the acquisition of effective coping skills. SIT has been already validated in clinical contexts, to help patients in facing particularly strenuous conditions (Amiri et al., 2017) and it has also been applied to cancer patients, showing its effectiveness in altering anxiety-related behaviors (Moore and Altmaier, 1981).

According to the results of a recent systematic review (Serino et al., 2014), the combination of a traditional SIT intervention with advanced technologies appears to be a promising clinical approach. In oncological care, Villani et al. (2013) showed the effectiveness of a mobile SIT intervention in reducing anxiety and improving active coping skills in oncology nurses.

As explained above, to target specifically the needs of aging breast cancer patients, we decided to set up and test this validated intervention on this population. Thus, we developed an eHealth intervention for older cancer patients based on a 2 weeks protocol consistent with the general SIT objectives: (1) increasing women’s knowledge about the emotional distress process, (2) developing emotion-regulation skills, and (3) helping women to apply the acquired coping skills in “real” contexts (which were simulated in this specific intervention).

The aim of the study was to test the efficacy of the e-health SIT intervention on emotional well-being, compared with a control group without intervention. Specifically, we expected an increase of adaptive emotion regulation strategies, defined as the “processes responsible for monitoring, evaluating, and modifying emotional reactions, especially their intensive and temporal features, to accomplish one’s goals (Thompson, 1994, pp. 27–28)”; and an increase of cancer-related well-being, with a specific focus on the emotional dimension of well-being, consistently with the focus of the intervention on improving emotional coping skills.

## Materials and Methods

### Participants

Patients were recruited primarily through direct patient contact with consecutively scheduled patients by two oncologists at two hospitals in Milan (ASST Rodhense and S. Giuseppe Multimedica Hospital). The study was proposed to all breast cancer patients fulfilling the following inclusion criteria: diagnosis of breast cancer with radical surgery; age over 55 years; negative staging for distant metastases; and suitability for adjuvant chemotherapy with anthracyclines and taxanes. Prior to analyzing data, we calculated the sufficient sample size needed to detect a medium effect size in our analyses. By convention, an *f*-value of 0.10 for effect size is considered small, 0.25 is medium and 0.40 is large. We used the software G^∗^Power and we found that 28 individuals were needed to provide 80% power to detect a medium effect size (*f* = 0.25) with a repeated measure design-within/between interaction (number of groups: 2; number of measurements: 3; correlation among repeated measures: 0.5).

By taking in consideration the clinical dimension, we decided to propose the study to forty patients to overcome drop-out at follow-up due to negative treatment-related side effects. Thus, the study was proposed to forty patients, but four gave up after the first meeting for personal reasons or hospital change and seven were not interested in participating (acceptance rate 72.5%; 29/40). Ultimately, 29 women (mean age = 62.76; *SD* = 6.19) voluntarily took part in the current study after signing giving informed consent. They were mostly married (79.3%; 23/29) and not employed (69%; 8/29 housewives and 12/29 retired). As concerns the educational level, 8 women had a primary school certificate, 8 women had a high school diploma, 10 women had a master degree and 3 women had obtained a post-master degree.

To investigate the effectiveness of the e-health SIT intervention, women were randomly allocated into two groups: the “e-Health Group” (EHG, *N* = 15) and the “Control Group” (CG, *N* = 14). Patients allocated to the control group received the traditional medical assistance offered by the hospitals, consisting of waiting for the first chemotherapy infusion. Psychological support before chemotherapy was not provided as usual care but only if requested by patients.

The study, conducted in compliance with the Helsinki Declaration (of 1975, as revised in 2008), was approved by the Ethics Review Board of the Department of Psychology of the Università Cattolica del Sacro Cuore of Milan (Italy).

### E-health Intervention

To support elderly breast cancer patients in their upcoming chemotherapy and offer them effective coping strategies, an e-health intervention based on SIT was specifically designed and delivered online through a dedicated website ^1^. Following the guidelines proposed by Meichenbaum (1985), the training was composed of three phases.

The first phase –*conceptualization*– aimed at helping patients to be aware of the nature of their own psychological stress. In the first meeting, during a face-to-face consultation with a psychologist, patients were helped to recognize the nature of their psychological stress related to the disease and the upcoming treatment. To facilitate awareness about the upcoming situation and its psychological impact, they experienced a live-video simulation of a chemotherapy session that they would receive within a few weeks. In particular, they were encouraged to reflect about their stress responses and the perceived threats and skills that could manifest in the different phases of the treatment. At the end of this first session, patients followed the intervention online at home for a period of 2 weeks; they were instructed on how to access the online intervention with personal account credentials. However, the psychologist’s contact was provided to all patients in case of personal difficulties during the treatment.

The e-health intervention comprised the other two phases of the traditional SIT, i.e., the *skills acquisition and rehearsal* (sessions 1–7) and the *application and follow-through* phase (sessions 8–10). The *skills acquisition and rehearsal* aimed at providing patients with effective coping skills and techniques to manage potential negative emotions that might occur during chemotherapy. Each session (25 min) was divided into two parts.

First, they were invited to watch live-video interviews with women who had gone through breast cancer experiences, to reflect upon their thoughts and emotions, to deeply understand chemotherapy side effects, and especially, to learn effective strategies through modeling to cope with physical and emotional changes. Specifically, interviews were focused on different crucial topics: women’s expectations, hopes and fears before starting chemotherapy; emotional experiences related to the disease and its impact on the quality of life; chemotherapy side effects, in particular hair loss, both from a practical and emotional point of view; the impact of the disease and therapies on several aspects of women’s life (physical, psychological, social, professional, etc.); potential activities that could be done during treatment to ameliorate side effects; suggestions and tips offering new perspectives that some women might find in this experience with social support.

Then, they were invited to experience relaxing videos with guided meditation audios to learn how to relax in disturbing moments. Included techniques were progressive muscle relaxation (Jacobson, 1938) and mindfulness-inspired strategies such as thought contemplation and detached mindfulness, aimed to help women to be aware of their thoughts and related emotions, and to accept their internal states (Kabat-Zinn, 2003). Even though there is considerable variability in mindfulness interventions being delivered in cancer care and reported benefits are related mostly to sub-clinical supportive care symptomology (Shaw et al., 2018), mindfulness strategies for women diagnosed with breast cancer during and subsequent to adjuvant treatment are recognized as effective short-term interventions in reducing affective difficulties, such as anxiety and depression (Haller et al., 2017).

**FIGURE 1:**
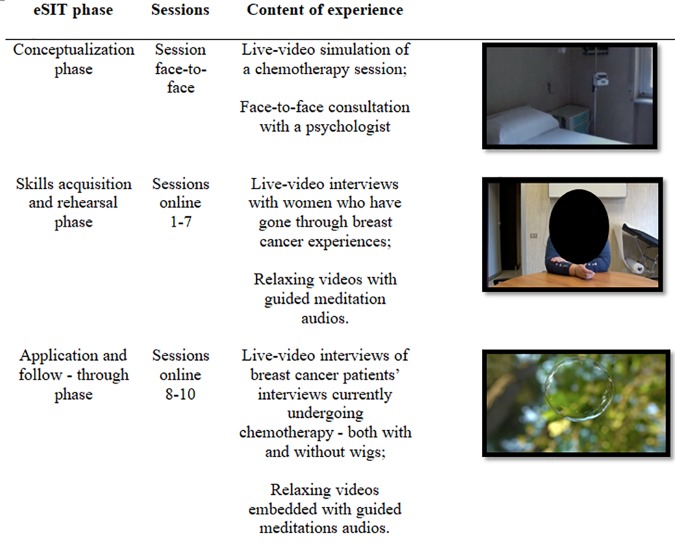
eSIT protocol.

Eventually, the aim of the *application and follow-through phase* was to expose women to the effects of their upcoming chemotherapy and help them to apply the acquired coping skills in a “real” context. Also, in this case, each session included two parts: first, participants were exposed to a series of live-video interviews of breast cancer patients’ currently undergoing chemotherapy – both with and without wigs. These videos also included tips and advice on how to cope with possible typical side effects occurring during the treatment. Then, with the help of relaxing videos embedded with guided meditation audios, participants were invited to apply the meditation exercises in a real context. Figure 1 provides a synthesized overview of the entire e-health protocol.

### Measures

#### Intervention Efficacy

**FIGURE 2:**
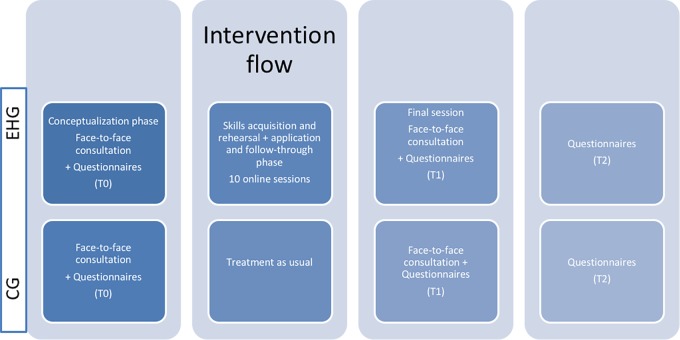
Intervention flow.

The e-health intervention was delivered with an online training lasting 2 weeks, while the CG only participated in the introductory and closing face-to-face consultations (see Figure 2).

To evaluate its efficacy in providing participants with effective emotional coping skills and in improving well-being related to disease, the following self-report questionnaires were administered to all participants at three time points: baseline (T0), after the 2 week intervention (T1), and 3 months after the end of the intervention (T2).

The Emotion Regulation Questionnaire (ERQ, Gross and John, 2003; Balzarotti et al., 2010) is a 10-item self-report questionnaire on a 7-point Likert scale [from 1 (strongly disagree) to 7 (strongly agree)] investigating individual differences in the use of the fundamental emotion regulation strategies. *Cognitive reappraisal* (6 items) refers to the ability to reinterpret the meaning of an emotional event to change its emotional impact (e.g., “When I want to feel more positive emotion, I change the way I’m thinking about the situation”; Cronbach’s alpha 0.775), while *emotional suppression* (4 items) refers to the tendency to inhibit the emotional expression elicited by a situation (e.g., “I control my emotions by not expressing them”; Cronbach’s alpha.746). The two scales were obtained by calculating the mean scores from the items, ranging from 1 to 7.

The Functional Assessment of Chronic Illness Therapy – Breast (FACT-B, Bonomi et al., 1996; Brady et al., 1997) is a self-administered questionnaire on a 5-point Likert scale that measures health-related well-being. The FACT-B comprises 29 general items that measure four subscales of quality of life: physical well-being (*PWB*, e.g., *“*I have a lack of energy,” reverse score item; Cronbach’s alpha 0.864), social well-being (*SWB*, e.g., *“*I get emotional support from my family*”*; Cronbach’s alpha 0.814), emotional well-being (*EWB*, e.g., “I feel nervous,” reverse score item; Cronbach’s alpha 0.624), and functional well-being (*FWB*, e.g., “I am able to work*”*; Cronbach’s alpha 0.651). Total score is obtained by summing individual subscale scores (FACT-General total score).

#### Emotional Changes and Intervention Acceptance

To investigate the fluctuations in the emotional experience during the online intervention, participants receiving the e-health intervention (EHG) were evaluated before and at the end of each online session about their level of anxiety and relaxation through a 7-point visual analog scale.

Actually, as the acceptance of ehealth technologies is a critical factor influencing their effective use and thus fostering the active role of patients in their healthcare, the intervention acceptance was measured. According to the technology acceptance model (TAM), which is one of the most influential models in explaining user acceptance of information technology (Davis, 1989), it is possible to hypothesize two fundamental factors: *perceived usefulness* and *perceived ease of use.* In adjunct, as analyzed by other studies (Villani et al., 2018), the *positive affective attitude* toward technologies also represents an important dimension of technology acceptance. Following this theoretical model, the intervention acceptance included five online *ad hoc* questions presented upon completion of the daily protocol investigating the pleasantness, the perceived utility of the contents and (only for meditation videos) the perceived easiness of the proposed exercises.

To monitor whether participants were accessing any other forms of psychosocial intervention during the weeks of intervention and until the follow up assessment, we added the following question to the T2 assessment: “Have you received a psychological support (privately or through the referral hospital’s facilities) since you agreed to participate in this study?”

## Results

We compared the E-Health Group (EHG) and the Control Group (CG) at the baseline in order to detect any difference in the considered variables at the beginning of the study. Independent sample *t*-tests indicated that the two groups did not differ either in their emotion regulation strategies (ERQ - cognitive reappraisal: *t*(27) = 0.928, *p* = 0.361; emotional suppression: *t*(27) = -1.156, *p* = 0.258), or in their wellbeing related to the cancer [FACT-B – PWB: *t*(27) = 1.587, *p* = 0.124; SWB: *t*(27) = 1.669, *p* = 0.107; EWB: *t*(27) = 0.695, *p* = 0.493; FWB: *t*(27) = 0.979, *p* = 0.336; FACT-General Total Score: *t*(27) = 1.802, *p* = 0.083].

Table 1 provides a detailed overview of the descriptive statistics of considered variables at three time points [baseline (T0), after the 2 week intervention (T1), and 3 months after the end of the intervention (T2)] divided for the two groups.

**Table 1:** Descriptive data for EQR and FACT-B questionnaires.

			Mean	St. Dev.	Min.	Max.
ERQ	Emotional suppression	T0	EHG	2.93	1.80	1.00	5.50
			CG	3.63	1.38	1.50	6.00
		T1	EHG	2.91	1.68	1.00	5.50
			CG	3.79	1.39	1.00	5.75
		T2	EHG	2.33	1.12	1.00	4.50
			CG	4.15	1.45	1.25	6.50
	Cognitive reappraisal	T0	EHG	4.99	1.19	2.20	6.80
			CG	4.53	1.46	1.40	6.20
		T1	EHG	5.11	1.09	3.40	7.00
			CG	4.59	1.79	1.00	7.00
		T2	EHG	5.43	1.43	2.20	7.00
			CG	4.88	1.24	2.80	7.00
FACT-B	Physical wellbeing	T0	EHG	24.40	2.56	19.00	27.00
			CG	21.43	6.76	8.00	28.00
		T1	EHG	22.07	4.57	11.00	28.00
			CG	22.71	4.53	13.00	28.00
		T2	EHG	21.92	3.55	17.00	27.00
			CG	19.82	6.03	12.00	28.00
	Social wellbeing	T0	EHG	18.73	5.08	8.00	26.00
			CG	16.21	2.55	12.00	19.00
		T1	EHG	17.93	3.63	12.00	24.00
			CG	15.71	4.07	11.00	25.00
		T2	EHG	18.62	2.96	13.00	22.00
			CG	17.55	4.97	5.00	24.00
	Emotional wellbeing	T0	EHG	17.60	3.48	10.00	22.00
			CG	16.43	5.45	4.00	23.00
		T1	EHG	17.13	3.11	9.00	21.00
			CG	16.71	4.55	8.00	23.00
		T2	EHG	19.54	2.90	14.00	24.00
			CG	16.82	5.04	9.00	23.00
	Functional wellbeing	T0	EHG	14.13	4.34	5.00	20.00
			CG	12.64	3.82	8.00	21.00
		T1	EHG	13.73	4.30	6.00	20.00
			CG	12.43	6.00	5.00	23.00
		T2	EHG	14.31	3.28	9.00	21.00
			CG	13.45	6.59	3.00	24.00
	FACT-B general	T0	EHG	74.87	10.36	51.00	88.00
			CG	66.71	13.86	40.00	88.00
		T1	EHG	70.87	9.78	48.00	83.00
			CG	67.57	12.75	46.00	89.00
		T2	EHG	74.38	9.11	60.00	91.00
			CG	67.64	17.18	32.00	89.00

Three months after the end of the intervention (T2), only 3 women (2 of the EHG group and 1 from the CG) declared that they received a psychological support (all of them from the referral hospital’s facilities), while 15 women (8 of the EHG and 7 of the CG) did not. This very low participation in psychological consultations by women involved in the study was not considered in the subsequent analyses aimed to test the effectiveness of the intervention.

### Protocol Efficacy

We tested the efficacy of the e-health intervention on emotion regulation and well-being related to cancer. To do this, a series of repeated measures ANCOVAs was conducted, with the baseline (T0) measure of the different variables as covariate, the factor Time (with two levels: T1 and T2) as within subject variable, and the Factor Group (EHG vs. CG) as between subject variable (see Table 2 for the ANCOVA statistical values).

**Table 2:** Repeated measures ANCOVAS results.

			*F*	*df*	*p*	η^2^
EQR	Emotional suppression (ES)	ES (T0)	22.25	1,19	0.003	0.38
		Group^∗^Time	5.5	1,19	0.03^∗^	0.23
	Cognitive reappraisal (CR)	CR (T0)	9.2	1,19	0.07	0.33
		Group^∗^Time	0.01	1,19	0.92	0.001
FACT-B	Physical wellbeing (PWB)	PWB (T0)	15,18	1,21	0.001	0.42
		Group^∗^Time	2.95	1,21	0.1	0.12
	Social wellbeing (SWB)	SWB (T0)	5.56	1,21	0.03	0.21
		Group^∗^Time	1.11	1,21	0.3	0.05
	Emotional wellbeing (EWB)	EWB (T0)	52.86	1,21	0.000	0.72
		Group^∗^Time	5.83	1,21	0.03^∗^	0.22
	Functional wellbeing (FWB)	FWB (T0)	13.5	1,21	0.01	0.39
		Group^∗^Time	0.64	1,21	0.43	0.03
	FACT-B general (FACT-G)	FACT-G (T0)	23.31	1,21	0.000	0.53
		Group^∗^Time	0.99	1,21	0.33	0.05

*^∗^ Statistically significant.*

**FIGURE 3:**
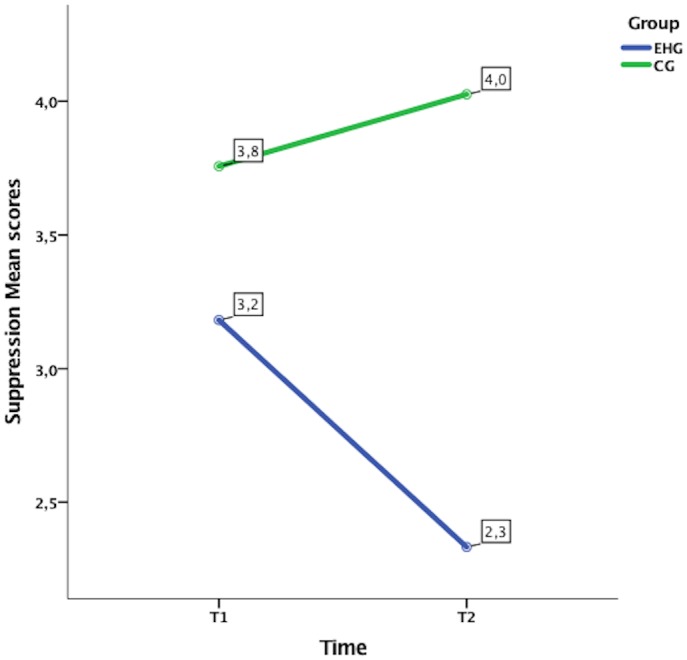
Emotional Suppression significant interaction effect time × group from ANCOVA analysis.

Concerning the emotion regulation strategy, as measured by ERQ, the initial level of emotional suppression was significantly related to the level of emotional suppression in the post-treatment (T1) and in the follow-up (T2). Furthermore, after controlling for the effect of the initial level of emotional suppression, the interaction Time × Group was also significant. Looking at the marginal means estimated by the model (see Figure 3), it is evident that in the EHG the emotional suppression decreased from T1 to T2 (T1 = 3.18; T2 = 2.33), whereas in the CG the scores were almost the same at the two time points (T1 = 3.76; T2 = 4.03). The initial scores of cognitive reappraisals were significantly related to the scores of the same variable in T1 and T2. Though, after controlling for the initial level of cognitive reappraisal, the interaction Time × Group was not significant, indicating that the two groups did not change differently from each other in that strategy over time.

**FIGURE 4:**
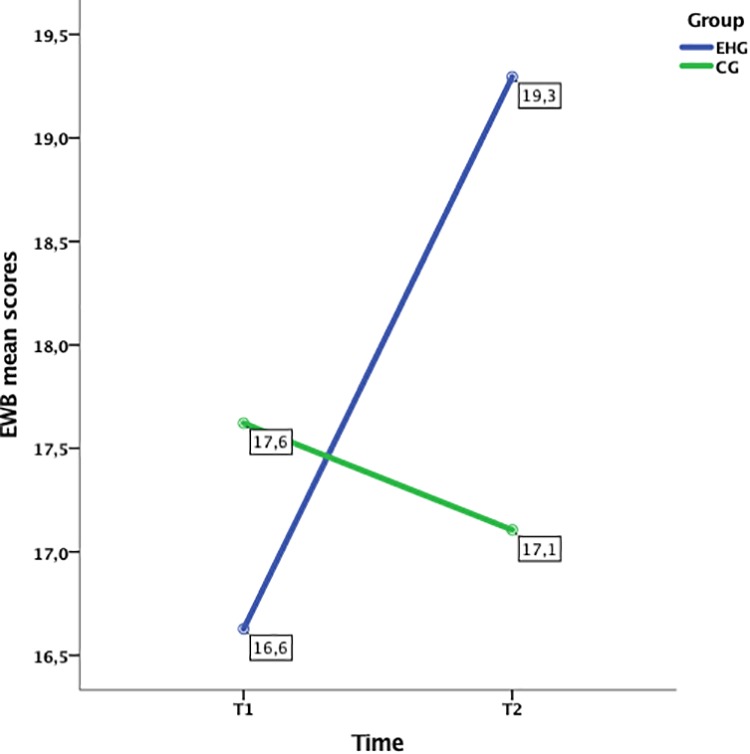
Emotional Well-being significant interaction effect time × group from ANCOVA analysis.

The analyses of the FACT-B evidenced that all the covariates significantly affected the scores of the correspondent variables in T1 and T2. However, only the EWB scores appeared significantly different over time in the two groups, after controlling for the initial EWB score (see Figure 4). The marginal means estimated by the model revealed that the EHG reported an increase in emotional wellbeing in the follow-up compared to the post-treatment (T1 = 16.63; T2 = 19.3), whereas the CG did not report any notable difference at the two time points (T1 = 17.62; T2 = 17.11).

### Emotional Changes and Intervention Acceptance

**FIGURE 5:**
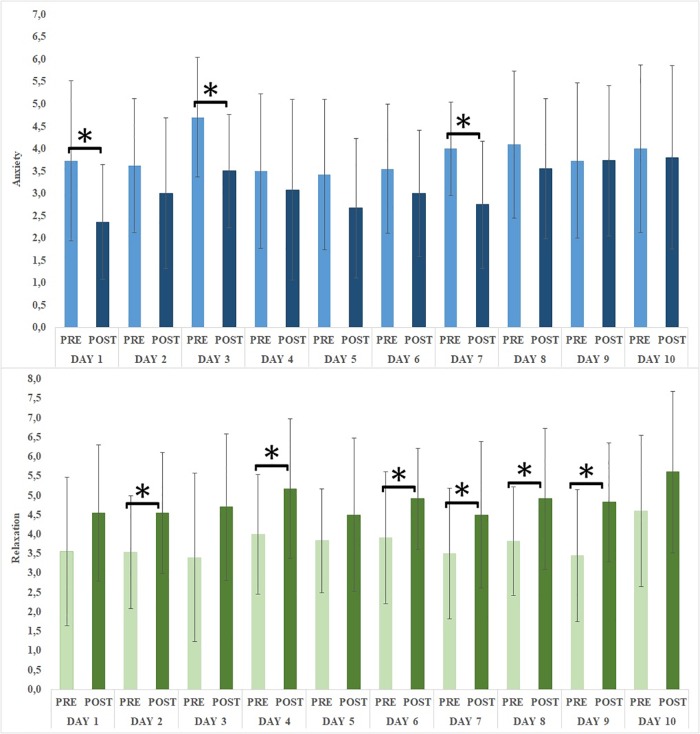
Emotional changes (anxiety and relaxation). Error bars: ±1 SD; ^∗^ significant changes

Changes in emotional states following the online intervention had been assessed by comparing VAS scores pre and post session, day by day (see Figure 5). Paired sample *t*-tests underlined a significant reduction of anxiety on days 1, 3, and 7 [respectively *t*(10) = 3.516, *p* = 0.006; *t*(9) = 2.571, *p* = 0.030; *t*(11) = 3.362, *p* = 0.006]. Furthermore, a significant increase of relaxation was reported on days 2, 4, 6, 7, 8, and 9 [respectively *t*(12) = -2.944, *p* = 0.012; *t*(11) = -2.880, *p* = 0.015; *t*(10) = -2.472, *p* = 0.033; *t*(11) = -2.872, *p* = 0.015; *t*(10) = -2.292, *p* = 0.045; *t*(10) = -3.012, *p* = 0.013]. No significant emotional changes were achieved in days 5 and 10 (respectively completed by 11 and 4 women).

The intervention acceptance was measured by averaging the scores of the five questions presented after completing the daily protocol, first across different days, and then across participants.

In relation to the skills acquisition and rehearsal phase (sessions 1–7), the pleasantness and usefulness of the video interviews were rated good (respectively, *M* = 5.18, *SD* = 0.37; *M* = 5.29, *SD* = 0.44) and also the pleasantness, usefulness and easiness of performing the exercises proposed within the video meditation and relaxation experience (respectively, *M* = 5.12, *SD* = 0.29; *M* = 5.16, *SD* = 0.19; *M* = 5.37, *SD* = 0.12). The same positive evaluation emerged in relation to the application and follow-through phase (sessions 8–10) from data assessing the pleasantness and the usefulness of the video interviews (respectively, *M* = 5.16, *SD* = 0.31; *M* = 5.27, *SD* = 0.24) and the pleasantness, usefulness and easiness of performing the exercises proposed within the video meditation and relaxation experience (respectively, *M* = 5.47, *SD* = 0.18; *M* = 5.24, *SD* = 0.21; *M* = 5.47, *SD* = 0.12).

## Discussion

Even if agreement about the experience of distress in breast cancer patients has not been found, some studies carried out in a similar cultural context – with an Italian sample – showed that, early on in the cancer trajectory, age can be considered one of the crucial precursor of patients’ distress (Chirico et al., 2015) and emotional distress is recognized as one of the principal indicators of vulnerability to long-term distress (Cook et al., 2018).

Breast cancer patients of all ages are uncertain as to whether or not they will experience and be able to cope with potential treatment-related side-effects such as severe nausea, vomiting, hair loss and tiredness. Thus, pre-chemotherapy counseling or education should help patients to increase their knowledge and develop active strategies to cope with the upcoming event. Coherent with this need and taking advantage of the opportunities offered by online intervention for these patients (McAlpine et al., 2015), this study developed and tested the efficacy of a 2 weeks ehealth intervention based on SIT (Meichenbaum, 1985) on emotion regulation and cancer-related well-being. This intervention was proposed after surgery and before chemotherapy and the ehealth group was compared with a control group without intervention.

To test protocol efficacy, self-report questionnaires were administered to all participants at baseline (T0), at the end of the 2 week intervention (T1), and 3 months after the end of the intervention (T2).

The ehealth group almost always completed the online sessions, with the exception of the last one (day 10). Results showed that after 2 weeks of ehealth intervention, patients did not achieve significant changes related to emotion regulation strategies, but they significantly reduced emotional suppression by 3 months after the end of the intervention. To understand these results, it is important to consider that, according to Gross and John (2003) 2 weeks is probably not enough time to modify patients’ emotion regulation strategies, which are generally built up over time and become a stable response to stress. Even if individuals are able to modify or improve these strategies, the result would not be immediately visible. However, after a sufficient time to be aware of their own internal processes, and after the beginning of chemotherapy treatment, breast cancer patients felt encouraged to express their emotions. This is an important result, as even if occasionally suppression is the best or even the only option to cope with the acute situation, it is recognized that this strategy is not helpful in reducing the experience of negative emotion that remains unsolved in the long term (Gross and John, 2003). Thus, emotional suppression has been shown to have a negative impact on a patient’s adjustment to cancer, whereas being able to express emotion can lessen distress and improve quality of life (Low et al., 2006). Furthermore, emotional expression can be seen as a proxy for obtaining social support; thus, it may engender positive social response. Specifically, Stanton and colleagues showed that women who coped through expressing emotions surrounding cancer had fewer medical appointments for cancer-related morbidities and decreased distress in the following three months, and those who perceived their social contexts as highly receptive reported an improvement in quality of life (Stanton et al., 2000).

The same trend has been found concerning cancer-related well-being. Specifically, the intervention significantly increased patients’ emotional well-being by 3 months after the end of the intervention. Despite some studies suggesting that older patients place great emphasis on antecedent emotion regulation and report great motivation to down-regulate negative emotions, probably assuming that aging is associated with a greater awareness of the inevitability of death (Scheibe and Carstensen, 2010), our study did not confirm this view: in fact, the control group did not improve their emotional strategies as time passed. This highlights both the importance of considering individual differences in older women (Boyle et al., 2017) and the value of designing interventions that facilitate more expression of emotion, which will allow help the patient accept and adapt to the progression of the disease (Moorey and Greer, 2002).

Specifically, the ehealth SIT intervention encouraged at least three adaptive strategies broadly associated with psychological well-being: acceptance, that is acknowledging that there are real issues and being aware of the negative experiences to come; emotional processing, in which the patient actively learns to understand the nature of the emotional experience; and emotional expression, which helps the patient to communicate emotional feelings, both in words and actions (Marroquín et al., 2017).

The emotional experience of patients also was monitored at a distance through the online compilation of the anxiety and relaxation questions. Results appeared positive, as we found that the emotional change due to the exposure to the online sessions helped patients in both reducing their anxiety and increasing their relaxation at some level. Furthermore, as the *application and follow-through* phase included live-video interviews of breast cancer patients currently undergoing chemotherapy – both with and without wigs – which could generate stress (Villani et al., 2013), it was encouraging to see that these sessions, followed by relaxing and meditation mindfulness experiences, helped patients to feel less stressed. To understand this positive outcome, we have to consider that the purpose of mindfulness is not to prevent negative experiences or change emotions, but rather to reframe the experience in a way that allows acceptance. By observing thoughts and feelings as mental events, patients have retrained negative thought patterns and reduced reactivity, thus fostering their sense of calm and well-being (Feldman et al., 2010; Hoge et al., 2015).

Finally, it is important to highlight that patients in the experimental group reported a good level of acceptance of the ehealth intervention. Live-video interviews were assessed as useful and pleasant. Meditation experiences were primarily assessed as very easy to perform, and also useful and pleasant. This result appears consistent with other studies showing that brief mindfulness meditation protocols can be successfully integrated in self-help interventions supported by new technologies and mobile apps, leading to a beneficial impact on stress, anxiety, depression, and well-being (Carissoli et al., 2015; Chittaro and Vianello, 2016; Spijkerman et al., 2016). While some studies argue that longer interventions are required to obtain positive outcomes (Baer et al., 2012), recent studies have suggested that very short mindfulness interventions can improve psychological well-being (Creswell et al., 2014), even when performed through a mobile app (Economides et al., 2018).

This result is similar to finding in other studies suggesting that Internet acceptance has reached an older age group, probably because patients older than 60 years are currently more familiar with the Internet than reported in previous studies (Santana et al., 2011), and indicates that the use of the Internet for health-related questions appears to be feasible for most patients (Drewes et al., 2016). In future years, with the increase of Internet access and computer literacy in society, we expect a growing interest in therapy assistance via digital health platforms. This trend, together with costs and cancer prevalence that are likely to increase as the population ages and increases, creates more pressure to develop new, less expensive treatments aimed to enhance patients’ self-management skills.

Before concluding, we have to underline some limitations of the study. First, the small sample size, related to the difficulties in recruiting elderly women undergoing chemotherapy, that did not include the patients’ starting level of distress. This issue may have reduced the ability to identify benefits of the ehealth intervention and future studies are encouraged to replicate the study with a wider sample and to assess the level of distress and the need of intervention as inclusion criteria. Second, the lack of an active control group, which is difficult to include considering that psychological interventions are usually offered during the chemotherapy treatment and not before. Other studies have proposed using the waiting list group, but this methodology did not appear suitable for this case as patients started chemotherapy after the brief ehealth intervention. The integration of the first and second limitation did not allow us to draw the effectiveness of the ehealth intervention in a definitive way. Future studies could integrate a placebo group, to which to propose an Internet intervention with content not consistent with the disease, in order to verify the effectiveness of the this ehealth intervention. Third, another limitation was the short time period analyzed (from definitive diagnosis, after operation, to the first months of chemotherapy), leaving out important moments such as the end of treatment and follow-up.

## Conclusion

To conclude, designing and developing eHealth interventions as part of regular care for breast cancer patients of all ages represents a challenge for future interventions. Specifically, immersive technology could be implemented in other phases of the treatment, such as during chemotherapy, to help women with breast cancer to facilitate a reduced time perception through distraction (Schneider et al., 2011) or to increase their knowledge and anxiety management (Jimenez et al., 2018). Furthermore, one possible way forward may be to view online interventions as an important step in universal psychosocial care within a tiered model of care. For example, distressed patients could be offered a low-cost self-managed online program such as this eSIT protocol and then transitioned to other more in-depth care models (such as psychotherapy treatment) if their distress remains unresolved (Chambers et al., 2018).

## Author Contributions

DV developed the study concept. CC was involved in the data collection. DV, CR, and SS performed the data analysis and interpretation, wrote the first draft of the manuscript. All authors were involved in the critical revision of the manuscript for important intellectual content, approved the final version of the manuscript for submission, and contributed to the study design.

## Conflict of Interest Statement

The authors declare that the researchwas conducted in the absence of any commercial or financial relationships that could be construed as a potential conflict of interest.

## Footnotes

1 www.conilsenodipoi.it
